# Fournier’s gangrene: our experience with 50 patients and analysis of factors affecting mortality

**DOI:** 10.1186/1749-7922-8-13

**Published:** 2013-04-01

**Authors:** El Bachir Benjelloun, Tarik Souiki, Nadia Yakla, Abdelmalek Ousadden, Khalid Mazaz, Abdellatif Louchi, Nabil Kanjaa, Khalid Ait Taleb

**Affiliations:** 1 Department of surgery, University hospital Hassan II, Fez, Morocco; 2 Department of Anesthesiology and Intensive Care, University hospital Hassan II, Fez, Morocco

**Keywords:** Fournier’s gangrene, Mortality, Outcome analysis

## Abstract

**Introduction:**

Fournier’s gangrene is a rare, rapidly progressive, necrotizing fasciitis of the external genitalia and perineum. Case series have shown a mortality rate of 20% to 40% with an incidence of as high as 88% in some reports. In this study we aimed to share our experience in the management of Fournier’s gangrene and to identify risk factors that affect mortality.

**Methods:**

The medical records of 50 patients with Fournier’s gangrene who presented at the University Hospital Hassan II of Fez from January 2003 to December 2009 were reviewed retrospectively to analyze the outcome and identify the risk factors and prognostic indicators of mortality.

**Results:**

Ten males and five females were enrolled in the study. The mean age was 54 years (range 23–81). The most common predisposing factor was diabetes mellitus (34%). E. coli was the most frequent bacterial organisms cultured. All patients were treated with a common approach of resuscitation, broad-spectrum antibiotics, and wide surgical excision. The mortality rate was 24%. The advanced age, renal failure on admission, extension of infection to the abdominal wall, occurrence of septic shock and need for postoperative mechanical ventilation are the main prognostic factors of mortality. In multivariate analysis, none of these variables is an independent predictor of mortality.

**Conclusions:**

Fournier’s gangrene is still a very severe disease with high mortality rates. Early recognition of infection associated with invasive and aggressive treatment is essential for attempting to reduce these prognostic indices.

## Introduction

Fournier’s gangrene (FG) is a rare, rapidly progressive, fulminant form of necrotizing fasciitis of the genital, perianal and perineal regions, which may extend up to the abdominal wall between the fascial planes [1]. It is secondary to polymicrobial infection by aerobic and anaerobic bacteria with a synergistic action [2-4]. The cause of infection is identifiable in 95% of cases, mainly arising from anorectal, genito-urinary and cutaneous sources [5]. Predisposing factors such as diabetes and Immunosuppression lead to vascular disease and suppressed immunity that increase susceptibility to polymicrobial Infection. Diagnosis is based on clinical signs and physical examination. Radiological methods may help to delineate the extent of the disease but false negatives may happen. Dissemination of the disease was found to be a major determinant of patients’ outcomes in previous reports [6,7]. It may reflect the aggressiveness of the involved infectious agents or reflects the degree of patients’ immunosuppression. Several reports tried to evaluate the usefulness of diverse scoring systems. Fournier’s Gangrene Severity Index (FGSI) has become a standard for researchers, being routinely published in FG literature and is considered as a good predicting tool [8,9]. The mortality rate for FG is still high, at 20–50% in most contemporary series [10,11]. Fortunately, it is a rare condition, with a reported incidence of 1.6/100,000 males with peak incidence in the 5th and 6th decades. However, the incidence is rising, most likely due to an increase in the mean age of the population, as well as increased numbers of patients on immunosuppressive therapy or suffering from human immunodeficiency virus (HIV) infection, especially in Africa [12,13]. Early diagnosis, aggressive resuscitation of the patient, administration of broad-spectrum antibiotics and aggressive radical surgical debridement(s), are the key of successful treatment. In this study, we aimed to investigate patients with FG and to identify risk factors that affect mortality.

## Materials and methods

The medical records of 50 consecutive patients admitted to the University Hospital Hassan II of Fez, Morocco, General Surgery Department, with a diagnosis of Fournier’s gangrene during the 7-year period between January 2003 and December 2009 were retrospectively reviewed. The inclusion criteria included patients undergoing wide surgical excision of scrotal and/or perineal necrosis along with other involved areas with a postoperative diagnosis of Fournier’s gangrene. Excluded were patients who had a local superficial inflammation of the perianal or urogenital regions as they were treated in Urology Department. Mortality was defined as disease-related death during the hospital stay and survival was measured in days. The prognostic variables used in the outcome analysis were the patient’s age, female gender, history of diabetes, the interval between the onset of symptoms and the initial debridement, renal failure, need for postoperative mechanical ventilation and occurrence of septic shock. Statistical analysis was performed using SPSS® 10.0 for Windows®. Mortality was accepted as disease-related death during the hospitalization period. The correlation of prognostic variables and mortality were studied by univariate analysis using chi-squared test and Fisher’s exact probability test. Statistically significant variables were entered into multivariate regression analysis using logistic regression. *P* values were reported as the result of two-tailed testing and *P* values less than 0.05 were considered as statistically significant. The study was performed according to the declaration of Helsinki and approved by the Local Ethical Committee.

## Results

Of the 50 patients studied, 12 died and 38 survived; the overall mortality rate was 24%. There were 44 men and 6 women with a mean age of 48 ± 16.81 years (range 18–85 years). The survivors (mean age 44.36 + 16.05 years) were significantly younger than the non-survivors (mean age 57.5 + 19.24 years) (p < 0.001). Sex was not a factor affecting mortality, even if the mortality among women was slightly higher (33.33%) compared to men (29.41%), but it did not reach statistical significance (p = 0.14). The source of infection was identified in 72 percent of the patients. The commonest source of sepsis was the anorectum (Table 1). Twenty one patients had at least one comorbidity. Diabetes mellitus (DM) was the most common comorbidity associated with FG and was present in 17 patients (34%) at the time of admission. In 29 patients (58%), predisposing factors could not be identified. Diabetes mellitus was not a factor affecting mortality as the mortality rate among non-diabetic patients was higher (49%) than patient with DM (41%) (p = 0.3). Furthermore DM did not influence hospital stay or number of debridments (Table 2).

**Table 1:** Etiology in 50 patients with Fournier’s gangrene

**Etiology**	**Patients**	**%**
Anal Abscess	31	62
Thrombosed hemorrhoid	4	8
Strangulated inguinal hernia	1	2
Unknown	14	28

**Table 2:** Impact of diabetes on the outcome variables in patients with Fournier’s gangrene

	**Diabetic patients n =17**	**Non-diabetic patients n =33**	**p**
Number of debridements (median values)	2.5	1.8	0.08
Length of hospital stay (median values)	15	12	0.5
Fecal diversion	2/17 (11.76%)	3/33 (9.09%)	0.7

The most common symptoms at the time of admission were deterioration of the generally state (44%), perineal necrosis (92%), fever (60%), perineal or genital pain (76%), septic shock (22%). the average time of symptoms prior to referral to treatment was 11 days, ranging from 4 to 25 days.

Computer Tomography of the pelvis was performed in only 2 patients (4%).

Regarding the exams performed on admission, complete blood count showed the presence of a hyperleukocytosis (> 10.000/mm3) in 39 patients (78%). The degree of anemia was severe necessitating blood transfusion in 9 patients (18%). Renal failure on admission (blood urea >0.5 g/l) was higher among the patients who died when compared to the survival group (p < 0.001).

As for the location and extent of the injury, it was observed that FG was confined to the perineal area in 5 patients (10%), affecting the scrotum in 35 (70%) individuals. The gangrene extended to the abdominal wall in 9 patients (18%) and thorax in 1 patient (2%). It was found that the extension of the infection to the abdominal wall was a predictor of mortality (p < 0.003 ) (50% in the non survivors compared to 7% in the survivors). The most frequent bacterial organisms cultured from the wound sites were *Escherichia coli* (85.6%) and *Klebsiella* (40.5%). Before surgery, all patients underwent aggressive fluid resuscitation and were treated mostly with parenteral broad-spectrum triple antimicrobial agents, using a third-generation cephalosporin, an amino glycoside and metronidazole and received hemodynamic support when required. Mechanical ventilation, continuous monitoring, and inotropic support were applied when necessary in patients with cardiopulmonary failure due to sepsis. All patients underwent radical surgical debridement, ranging from 1 to 10 procedures, with an average of 2.5. Debridement consisted of excision of all necrotic tissue, cleansing with hydrogen peroxide, then saline and drainage. Along with the initial radical debridement, 5 patients (10%) underwent fecal diversion, with loop colostomy. Orchidectomy was carried out unilaterally for gangrenous testes in one patient (2%). It’s interesting to notice that mortality rate was 52.63% in the single-debridement group and 66.66% in repeated debridements; however, these rates were not significantly different (*p* = 0.08). Mechanical ventilation, due to sepsis was applied in 11 patients (22%). It was significantly higher in non survivor patients (91.6%) comparing to the survivors (0%) (p < 0.001). Patients had a median hospital stay of 21 (range, 4–66) days. The median hospitalization time (MHT) for the surviving patients was 26.00 days compared to 8.00 days for the non-survivors (P < 0.001).

As a result, evaluation of the outcome variables by univariate analysis demonstrated for statistically significant predictors of mortality, which were the advanced age, extension of the infection to the abdominal wall, renal failure and need of Mechanical ventilation (Table 3). However the presence of diabetes, female gender, interval between the symptoms and surgical intervention and repeated debridements did not appear as predictors of mortality. In the subsequent multivariate analysis, none of above studied variables was identified as independent predictors of mortality.

**Table 3:** Comparison of the patients’ characteristics between survivors and non-survivors

**Patient characteristics**	**Survivors n = 38**	**Non-survivors n = 12**	**p**
Age (years, mean ± SD)	44.36 ± 16.05	57.5 ± 19.24	<0.001
Duration of symptoms (days, median values)	11	11.3	0.83
Presence of Diabetes Mellitus	31.57%	41.66%	0.075
Extension of the infection to the abdominal wall	7%	50%	<0.003
Number of debridements (median values)	3.5	2.5	0.086
Renal failure	18.42%	83.33%	<0.001
Need of Mechanical ventilation	0%	91.6%	<0.0001

## Discussion

**Figure 1:**
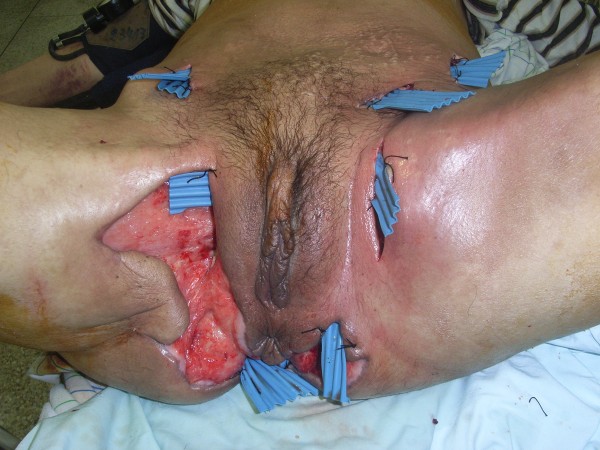
Fournier’s gangrene with extension to the abdominal wall.

Fournier’s gangrene, caused by synergistic aerobic and anaerobic organisms, is a life-threatening disorder in which infection of the perineum and scrotum spreads along fascial planes, leading to soft-tissue necrosis. This infectious was initially described by Baurienne in 1764 [14]. Before in 1883 Jean Alfred Fournier, French dermatologist described a syndrome of unexplained sudden onset and rapidly progressing gangrene in the penis and scrotum of 5 young men with no other pathology basis of sudden onset and rapid progression [15]. In its early reports Fournier’s gangrene was described as an idiopathic entity, but in most cases a perianal infection, urinary tract and local trauma or skin condition at that level can be identified [12]. The mortality rate for FG is still high, (20–50%) in most contemporary series [10,11], despite an increased knowledge of the etiology, diagnosis and treatment, and intensive-care techniques. The high mortality reflects both the aggressive nature of the infection and the destructive effects of accompanying predisposing factors. Several factors affecting the mortality were studied such as increasing age, primary anorectal infections, existence of diabetes, delay in treatment, evidence of systemic sepsis at presentation, extent and depth of involvement, a low haematocrit, a high leukocytosis and blood urea nitrogen, a high alkaline phosphatase and serum albumin, and many others [8-13,16-19]. These and other studied variables that influence the outcome of patients with FG, in large part, remains controversial. In this purpose, the FGSI was developed to help clinicians predict the outcome of patients with FG and remains an objective and simple method to quantify the extent of metabolic aberration at presentation in patients with FG. It has been validated in several reported studies [8,9,11,17]. The average age of the patients was 47.5 years, in most published series from 40.9 to 61.7 years [10,12]. In a population based study of 1641 patients, Sorensen *et al.* found that an increasing patient age was the strongest independent predictor of mortality (aOR 4.0 to 15.0, p <0.0001) [12]. Our results are in keeping with the study of Sorensen et al. as the survivors were significantly younger than the non-survivors in our series. With regard to gender, the male predominance is reported in 96%, so the female was present only in 4% [10,12]. Czymek *et al.*, compared mortality between male and female in a series of 38 patients (26 M vs 12 F). Authors found that mortality is significantly higher among female (50% F vs 7.7% M, p = 0.0011) [18]. We could not confirm this result, as female gender did not appear as predictor factor of mortality in our study (Table 4). Numerous factors have been implicated at the onset of FG, in particular, those involving the immune system [19-22]. Diabetes mellitus was the most reported co-morbid disease associated with this pathology. Some authors estimate the prevalence of DM among FG patients between 50 and 70 percent [23-25]. Despite of being a risk factor for FG and associated with a more progressive and fatal outcome (decreased phagocytic and intracellular bactericidal activity and neutrophil dysfunction), most reported studies along with our have failed to demonstrate the influence of DM on outcomes in FG [26-28]. It is also suggested that renal failure on admission might be a noticeable factor for the prediction of the mortality rate [8,29]. Among many laboratory parameters studied in FG, Clayton *et al.,* reported that only a level of blood urea >0.5 g/l on admission was statistically significant for mortality [30]. In our study we also found that renal failure on admission is significantly higher in non survivors. Few articles have highlighted the poor prognosis of FG in patients with a delay between time of presentation and treatment. This factor has been reported in a study by Jeong *et al.*, as a predictor of mortality [6]. Along with other studies, we did not find delay this to be a major predictor of mortality [31,32]. The extension of the disease and the mortality rate are controversial themes in the literature. Some studies have reported that the spread of the disease is related to a higher death rate, while other studies report that the extension of the gangrene does not relate to a poorer prognosis [30,33]. In this field, extent to abdominal wall (Figure 1) has been reported to be directly related to mortality [22,34,35], which was confirmed in our series. Ultimately, occurrence of septic shock and need for postoperative mechanical ventilation, have been demonstrated as a powerful (even late) factors of mortality [8,9,24,36]. Furthermore, Yanar *et al.* found that the presence of sepsis was as the only significant independent risk factor for mortality in FG [3]. Our results join those reported in literature, although in multivariate analysis, these parameters have been not identified as independent predictors of mortality. Finally we acknowledge that our study has important limitations. Data collection was retrospective, the patient cohort is small, we focused on some variables but surely dismiss others not less important, we did not have access to important clinical and laboratory data so that we could not use and evaluate the performance of the Fournier's Gangrene Severity Index.

**Table 4:** Mortality among male and female in different series

**Series**	**Number of cases**	**Male**	**Female**	**p**
Jarboui *et al.*, 2007 [24]	35	24%	25%	<0.05
Cyzmek *et al.*, 2010 [18]	51	7,7%	50%	0.0011
Garcia Marin *et al.*, 2011 [23]	34	30%	0%	0.273
Ours series	50	29,41%	33,33%	0.14

## Conclusions

Fournier’s gangrene is still a very severe disease with a high mortality rate. The advanced age, renal failure on admission, extension of infection to the abdominal wall, occurrence of septic shock and need for postoperative mechanical ventilation are the main prognostic factors of mortality. Early recognition of infection associated with invasive and aggressive treatment is essential for attempting to reduce these prognostic indices.

## Competing interests

The authors declare that they have no competing interests.

## Authors' contributions

(1) BEB have made substantial contributions to conception, bibliography and drafting the manuscript. (2) TS have been involved in statistical analysis and interpretation of data. (3) NY have been involved in acquisition of data and bibliography research (4) AO and (5) KM have been involved in revising it critically for important intellectual content. (6) AL and (7) NK have been involved in the conception of the study. (8) AK has given final approval of the version to be published. All authors read and approved the final manuscript.
